# Effects of *Anchomanes difformis* on Inflammation, Apoptosis, and Organ Toxicity in STZ-Induced Diabetic Cardiomyopathy

**DOI:** 10.3390/biomedicines8020029

**Published:** 2020-02-08

**Authors:** Toyin D. Alabi, Novel N. Chegou, Nicole L. Brooks, Oluwafemi O. Oguntibeju

**Affiliations:** 1 Phytomedicine & Phytochemistry Group, Oxidative Stress Research Centre, Department of Biomedical Sciences, Faculty of Health and Wellness Sciences, Cape Peninsula University of Technology, Bellville 7535, South Africa; toyudoc@yahoo.com; 2 DST-NRF Centre of Excellence for Biomedical Tuberculosis Research, South African Medical Research Council Centre for Tuberculosis Research, Division of Molecular Biology and Human Genetics, Faculty of Medicine and Health Sciences, Stellenbosch University, P.O. Box 241, Cape Town 8000, South Africa; novel@sun.ac.za; 3 Faculty of Health and Wellness Sciences, Cape Peninsula University of Technology, Cape Town 8000, South Africa; brooksn@cput.ac.za

**Keywords:** *Anchomanes difformis*, apoptosis, cardiomyopathy, diabetes, glibenclamide, inflammation, oxidative stress

## Abstract

Persistent hyperglycemia is known to cause enhanced generation of reactive oxygen species in diabetes. Several inflammatory cytokines are induced by oxidative stress, and their release also leads to increased oxidative stress; this makes oxidative stress one of the important factors in the development of chronic inflammation and other immune responses. These have been implicated in the development of diabetic complications such as nephropathy and cardiomyopathy. *Anchomanes difformis* has been shown to possess antioxidant and anti-inflammatory potentials. The present study investigated the immunomodulatory potential and the antiapoptotic ability of *Anchomanes difformis* to ameliorate heart toxicity and injury in type II diabetes. Two weeks of fructose (10%) administration followed by single intraperitoneal injection of streptozotocin (40 mg/kg) were used to induce type II diabetes in male Wistar rats. Leaf extract (aqueous) of *Anchomanes difformis* (200 and 400 mg/kg) was administered orally for six weeks. Blood glucose concentrations and body weights before and after interventions were determined. Interleukin (IL)-1β, IL-6, IL-10, IL-18, monocyte chemoattractant protein 1 (MCP-1), and tumor necrosis factor alpha (TNFα) were measured in the heart homogenates. Catalase (CAT), superoxide dismutase (SOD), total protein, oxygen radical absorbance capacity (ORAC), ferric reducing antioxidant power (FRAP), thiobarbituric acid reactive substances (TBARS), and heart-type fatty acid-binding protein (H-FABP) levels were determined. Expressions of transcription factors (Nrf 2 and NFkB/p65) and apoptotic markers were also investigated in the heart. *Anchomanes* *difformis* administration reduced pro-inflammatory cytokines, increased anti-inflammatory markers, and enhanced antioxidant defense in the heart of diabetic treated animals. *Anchomanes* *difformis* is a new, promising therapeutic agent that can be explored for the treatment of pathological conditions associated with immune responses and will be a useful tool in the management of associated diabetic complications.

## 1. Introduction

**Figure 1:**
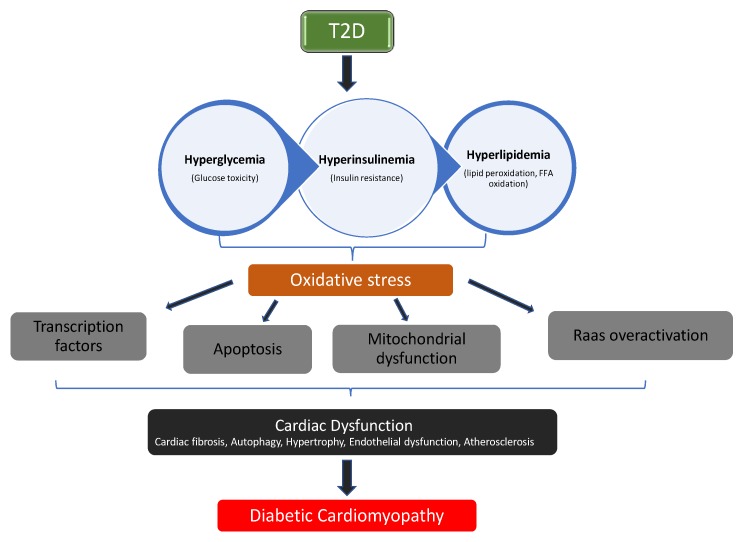
The pathophysiological pathway in the progression of diabetic cardiomyopathy (DCM).

Constant hyperglycemia—a major characteristic of diabetes mellitus (DM)—has been linked to overproduction of reactive oxygen species (ROS). Excessive generation of ROS activates cytokines production, apoptotic proteins, and transcription factors, resulting in chronic inflammation and increased apoptosis, an underlying factor in the development of diabetic complications [1]. Evidence suggests that oxidative damage, pro-inflammatory responses, and apoptosis are key players in pathological conditions relating to diabetic cardiomyopathy [2,3]. Diabetic cardiomyopathy (DCM) is a pathological condition of the heart observed in subjects with diabetes, and it is independent of other cardiovascular pathologies such as hypertension, ischemic heart injury, coronary artery disease, and congenital heart diseases. DCM is a major cause of heart failure [4,5]. The progression of diabetes mellitus to diabetic cardiomyopathy involves the interplay of several mechanisms: oxidative damage, inflammation, apoptosis, mitochondrial dysfunction, and overactivation of Raas (Renin-angiotensin aldosterone system), as illustrated in Figure 1 [6,7].

Inflammation is a form of defense and protection from infections and tissue damage; however, the uncontrolled regulation of immune responses can lead to excessive tissue damage [8,9]. Pro-inflammatory cytokines with other indices of inflammation have been shown to be elevated in the hearts of type II diabetes (T2D) subjects [10,11]. Diabetes triggers lower levels of systemic inflammation in the cardiomyocytes as an early response to myocardial injury due to the overproduction of mitochondrial ROS. This systemic inflammation triggers the recruitment of leukocytes and causes the secretion of pro-inflammatory cytokines and chemokines such as interleukin (IL)-1β, IL-6, and tumor necrosis factor alpha (TNF-α) [6,12]. Further exposure to high concentrations of glucose results in increased production of advanced glycation end products (AGEs). AGEs are regulators of endothelial cell permeability and migration of monocytes and ultimately activate nuclear factor kappa-light-chain enhancers of activated B cells (NFkB) [13]. NFkB primarily stimulates the expression of more pro-inflammatory cytokines (TNF-α, IL-6, IL-18) in the heart, which are associated with hypertrophy, fibrosis, and left ventricular dysfunction. This cascade of reactions is repeated severally, leading to a sustained immune response and thereby causing further injury and cardiomyopathy [7,14].

The significance of maintaining homeostasis in multicellular organisms is pivotal and having a balance between cell death and proliferation plays a key role in homeostasis. Cell death could occur in the form of necrosis, apoptosis, or autophagy. Apoptosis is a programmed cell death that serves as a natural barrier against uncontrolled proliferation of cells and prevents injury that may be caused by damaged or stressed cells [15]. Excess cellular levels of ROS cause damage to biomolecules, membranes, and organelles, which activates cell death processes such as apoptosis [16]. However, increased apoptosis has been implicated in the development of diabetic complications (DCM). Recently, Nunes and co-workers reported the influence of monocyte chemoattractant protein 1 (MCP-1 on apoptosis in cardiomyocytes. This substantiates the link between inflammation, apoptosis, and DCM [17].

During increased oxidative stress, the body system augments its antioxidant capacities to combat elevated oxidative stress. The nuclear factor E2-related factor 2 (Nrf2) is a transcription factor that is associated with mitigating oxidative stress [18]. Nrf2 is activated in the cytosol to counteract increased oxidative stress and maintain homeostasis. This is done by enhancing the expression of AREs such as catalase (CAT), superoxide dismutase (SOD), GSH, and GPx that will mop up the reactive oxygen and nitrogen species. Keap1, which is bound to actin in the cytosol, interacts with Nrf2, promotes ubiquitination, and eventually degrades Nrf2. This process is rapid, making the half-life of Nrf2 around 13–21 min [19,20]. This, however, keeps Nrf2 at relatively low basal levels. Conversely, during increased intracellular ROS, which leads to cellular stress, Keap1 loses its effectiveness in degrading Nrf2. This extends the half-life of Nrf2 to about 100–120 min, thereby stabilizing Nrf2 levels [19,20]. Nrf2 then enters the nucleus and triggers the transcription of AREs and other cytoprotective genes [21]. Investigation on the protective role of Nrf2 reported its significance in the regulation of inflammation and apoptosis. Nrf2 is involved in enhancing the expression of survival inflammatory markers, which in turn suppresses pro-apoptotic proteins. Hematopoietic stem cells with their Nrf2 gene knocked off showed increased oxidative stress, apoptosis, and reduced expression of pro-survival genes [22]. It was recently established that Nrf2 directly inhibits the transcription of pro-inflammatory genes coding for pro-inflammatory proteins (IL-1α, IL-1β, IL-6), therefore ameliorating increased inflammation and apoptosis [23].

Streptozotocin (STZ) is a glucose analogue that is cytotoxic to the beta cells of the pancreas. It is a diabetogenic compound that is produced naturally in *Streptomyces achromogenes,* a soil bacterium. The effect of STZ administration is usually seen within three days depending on the dosage. STZ exhibits its selective toxicity on beta cells in rats by DNA fragmentation of the beta cells and causes death, leading to diabetic conditions that further progress to diabetic complications if uncontrolled [24]. High dosage of STZ has been reported to result in complete destruction of the beta cells, a model of type I diabetes. Recent studies report the effective use of low doses of STZ to induce insulin resistance, a model of type II diabetes (T2D) [25,26,27]. Administration of 10% fructose for two weeks followed by 40 mg/kg BW of STZ was demonstrated by Wilson and Islam to cause partial destruction of the beta cells and insulin resistance in rats, which are typical of T2D [28].

*Anchomanes difformis* (AD) is a plant with numerous ethno-botanical uses in Africa for conditions such as inflammation, diabetes, asthma, microbial infections, pain, ulcerations, and gastrointestinal disturbances. Some of these folkloric uses have been scientifically established, while others are still indigenous claims [29]. The anti-inflammatory ability of the leaf and the rhizome extracts of AD was revealed by its inhibitory activity on histamine and serotonin, which are mediators in the initial phase of acute inflammation. AD showed more anti-inflammatory potential than the standard drug used, aspirin [30]. Similarly, Adebayo and colleagues also demonstrated the anti-inflammatory property of AD. The plant inhibited oedema (paw volume) in raw-egg albumin induced inflammation in chicks [31]. Studies show that AD is effective against hyperglycemia in alloxan-induced diabetes [32,33]; however, the potential of AD against inflammation and apoptosis in diabetic mellitus has not been explored. This study therefore investigates the anti-inflammatory and the anti-apoptotic ability of AD leaves extract (aqueous) on increased inflammatory response and cell death in STZ-induced diabetic cardiomyopathy in male Wistar rats. We carried out phytochemical characterization and profiling of six different extracts of AD, and 32 compounds were identified. Furthermore, antioxidant capacities of the extracts were measured using oxygen radical absorbance capacity (ORAC), ferric reducing antioxidant power (FRAP), and TEAC assays, and the aqueous extract exhibited the highest antioxidant capacity [34], hence its choice for this study. Aqueous extract of AD contains phytochemicals such as quercetin, phloridzin, kaempferol, rutin, and chlorogenic acids, among others, which are active against hyperglycaemia, oxidative stress, inflammation, and apoptosis [34,35,36]. The wide range of biological properties exhibited by compounds present in AD has necessitated further investigation into its potentials against diabetic cardiomyopathy.

## 2. Materials and Methods

### 2.1. Chemicals and Reagents

STZ was purchased from Biocom Africa, South Africa. Heart-type fatty acid-binding protein (H-FABP) (Cat. No: FB 4025) was obtained from Randox Laboratories (Johannesburg, South Africa). The interleukins and TNF-α were supplied by Biorad, while MCP-1 was supplied by Merck (South Africa). Anti-Nrf2, Anti-NFkB/p65, Anti-Bcl2, Anti-Caspase-3, Anti-mouse IgG H&L, and Anti-rabbit IgG H&L were bought from Biocom (Johannesburg, South Africa). Bicinchoninic Acid Protein Assay kit was purchased from Thermo Fisher Scientific, Johannesburg, South Africa.

### 2.2. Plant Preparation

The harvested leaves of *A. difformis* were authenticated (LUH6623), and a specimen was deposited at the herbarium, University of Lagos, Nigeria. The leaves were dried under shade, blended, and de-fatted with n-hexane. Aqueous extracts from the dried leaves of AD were extracted via cold extraction (2–8 °C). The extract was pulverized and stored at −20 °C for further analysis.

### 2.3. Ethical Approval

This study was approved by the Faculty of Health & Wellness Sciences Research Ethics Committee (REC) of the Cape Peninsula University of Technology, Bellville, South Africa (CPUT/HW-REC 2016/A4 (2016). It was also approved (REF.04/17) by the Ethics Committee for Research on Animals at the South African Medical Research Council (SAMRC), South Africa, where the animal experiment was performed.

### 2.4. Animals

Wistar rats (male) weighing approximately 180 ± 10 g (male) were obtained from Stellenbosch University (animal facility), South Africa. The animals were acclimatized for 3–4 weeks and housed at the Primate Unit & Delft Animal Centre (PUDAC), SAMRC, Cape Town. Housing conditions were controlled: humidity between 45% to 55% and temperature between 22 °C to 26 °C. Standard rat chow (SRC) and water was fed to all the rats ad libitum, and they were exposed to a normal photo period (12 h dark/12 h light). Animal handling, care, and other procedures were done in accordance with the standard operating procedure of SAMRC PUDAC (SOP No: 2016-R01), which conforms to the revised South African National Standard for the Care and Use of Animals for Scientific Purposes (South African Bureau of Standards, SANS 10386, 2008).

### 2.5. Experimental Design

**Figure 2:**
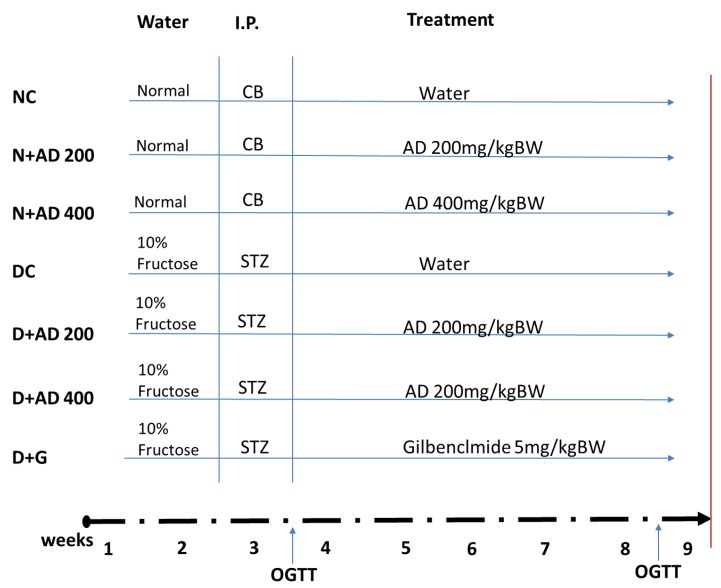
Experimental design. Animals were randomly assigned into 7 groups (*n* ≥ 8); 14 days of administration of 10% fructose preceded a single-dose injection of Streptozotocin (STZ) (40 mg/kg). After 5 days, animals with fasting blood glucose of 15 mmol/L or greater were considered diabetic. OGTT was conducted to confirm insulin resistance. Normal rats were administered the vehicles—water and citrate buffer (CB)—accordingly. Treatment commenced immediately for 42 days via oral gavage. Animals were euthanized on the 43rd day (red bar).

Sixty-four male Wistar rats with weights ranging from 270–300 g were used for this study. The rats were randomly assigned into seven groups with a minimum of eight rats in each group (8 rats in normal groups and 10 in diabetic groups), as summarized in Figure 2. Water served as the vehicle for fructose and AD administration, while citrate buffer was the vehicle for streptozotocin. Animals in group 1 served as the normal control (NC) and received the vehicle only. Animals in groups 2 and 3 were normal rats who received 200 and 400 mg/kg BW of AD aqueous extract only (N + AD 200 and N + AD 400), and these served as the treated control. Groups 4–7 consisted of animals that were placed on 10% fructose for 2 weeks followed by streptozotocin (STZ). Group 4 rats received vehicle only (DC), rats in groups 5 and 6 were given 200 and 400 mg/kg BW of AD aqueous extract (D + AD 200 and D + AD 400), respectively, while group 7 rats received 5 mg/kg BW of glibenclamide, an antidiabetic drug (D + G).

### 2.6. Blood and Tissue Collection

The rats were anaesthetized during euthanasia with 2% isoflurane per oxygen (1 L/min flow rate) via inhalation. Blood was collected into Z-serum clot activator tubes from the abdominal vein. Blood samples were centrifuged at 4000× *g* for 10 min at 4 °C. Aliquots of the supernatant were stored at −80 °C for biochemical analysis. During euthanasia, the heart was extracted and immediately washed in ice-cold phosphate buffered saline (PBS), dabbed, and weighed. This was then frozen using liquid nitrogen and later stored at −80 °C for further analysis.

### 2.7. Tissue Preparation

The heart samples for histological examination were fixed in 10% buffered formalin solution immediately. Samples that were to be used for immunofluorescence assays were fixed with a freezing media, froze in the liquid nitrogen, and further stored at −80 °C. Homogenization of the heart tissues was done subsequently for assays requiring tissue lysate. A 200 mg sample of the heart was homogenized on ice in 2 mL of 50 mM phosphate buffer with 0.5% triton and centrifuged at 10,000× gravitational force for 15 min at 4 °C. The supernatants were aliquoted and stored at −80 °C.

### 2.8. Determination of Organ Function and Toxicity Markers

The body weights of all the animals were measured weekly throughout the animal study. The relative heart weight was derived from the weight of the heart and the body weight of the same rat.

(1) $$Relative\;heart\;weight\;=\;\frac{Heart\;weight\;\left(g\right)}{Total\;body\;weight\;\left(g\right)}\;\times 100\%$$

Heart-type fatty acid-binding protein (H-FABP) was quantified in the serum, and the determination was carried out according to the standard operating procedure provided by the manufacturer. The principle of the assay was based on immunoturbidimetry.

### 2.9. Analysis of Antioxidant Status and Lipid Peroxidation Indices

Activities of catalase, superoxide dismutase, and levels of thiobarbituric acid reactive substances (TBARS) were assayed in the hearts of normal and diabetic rats. In addition, ORAC and FRAP were also measured to evaluate antioxidant capacity in the heart. The method of Ellerby and Bredesen [37] was used to determine catalase activities in the hearts, where the rate of conversion of hydrogen peroxide to water and oxygen by catalase was measured at 240 nm. SOD levels were also measured by the method of Ellerby and Bredesen [37], which measures the amount of the enzymes needed to exhibit dismutation of the superoxide radicals produced from the auto-oxidation of 6-hdroxydopamine (6-HD). TBARS levels were evaluated using the modified methods of Matsunami et al. and Wasowicz et al. [38,39]. ORAC levels were assessed according to the method of Prior et al., [40] while FRAP was measured using the method described by Benzie and Strain [41]. Total protein present in the heart was quantified using Bicinchoninic Acid Protein Assay kit, and manufacturer’s procedures were duly followed.

### 2.10. Estimation of Pro-and Anti-Inflammatory Biomarkers

Interleukins (IL)-1β, IL-6, and IL-10 were measured, and other chemokines including MCP-1 (monocyte chemotactic protein-1) and TNF-α (tumor necrosis factor-α) were also measured in the heart lysates of normal and diabetic rats. These inflammatory markers were measured using Bioplex Promagnetic bead-based assays (Bio-Rad Laboratories, Hercules, CA, USA) on the Bio-plex platform (Bio-Rad). Assays were performed according to the manufacturer’s instructions (BioRad and Merck Millipore, Burlington, MA, USA). Bead acquisition and analysis of median fluorescent intensities was done using Bio-Plex Manager software, (version 6.0, Hercules, CA, USA).

### 2.11. Evaluation of Apoptotic and Transcriptional Proteins Expression

To explore the effect of AD on Nrf2, NFkB/p65, Bcl2, and caspase-3 expression in increased oxidative stress and inflammatory process induced by hyperglycemia, we carried out an immunohistochemical fluorescence staining on the heart tissue. The process was carried out according to the manufacturer’s procedures.

### 2.12. Antibodies

Optimization of primary antibodies was carried out on the heart tissues to determine the optimal staining concentrations and conditions for each antibody. Single and double stains were done for each antibody at varying dilutions and diluents. The combination that showed the highest specificity and sensitivity on positive and negative control samples was considered as the optimal staining concentration. This was done with the help of an experienced pathologist. The details of each antibody used and their optimal concentration are presented in Table 1.

### 2.13. Tissue Preparation and Staining

Heart tissues were fixed with freezing media (Leica, Johannesburg, South Africa) and stored at −80 °C. Then, 10 µm of the tissues were sectioned using a Leica CM 1860 UV Cryostat and permeabilized with PBS plus 0.025% Triton X-100 (PBS-T). Tissues were blocked with 10% normal goat serum in PBS containing 5% bovine serum albumin (BSA) for 2 h. Incubation with respective primary antibodies (see details in Table 1) was done overnight at 4 °C, followed by 2 washes in PBS-T. Tissues were incubated with secondary antibodies for 1 h at room temperature in the dark and washed thrice with PBS. Mounting was done using Dako mounting medium (Agilent Technology Inc., Johannesburg, South Africa). The argon multiline laser at 488 nm and the DPSS 561–10 laser at 561 nm was used to excite the Alexa Fluor 488 (green) and Alexa Fluor 594 (red), respectively.

### 2.14. Imaging

Images were acquired on a Zeiss LSM780 ELYRA PS1 super-resolution, confocal microscope with a 10 x/0.3 M27 objective (EC “Plan-Neofluar”). Zen 2.6 imaging software (blue and black edition, Zeiss Germany) was used for image analysis and to obtain mean fluorescent intensities (MFI) on 4 images acquired in each experimental condition. This was repeated thrice.

### 2.15. Data Analysis

Values were expressed as mean ± standard error of mean (SEM). Statistical analysis of results was performed using one-way or two-way analysis of variance (ANOVA) to find differences between groups. Bonferroni test was used for all pair-wise comparisons. Differences (*F* values) were considered statistically significant at *p* values less than 0.05. All statistical calculations were done using GraphPad Prism Version 5.00 for Windows, GraphPad Inc., San Diego, CA, USA.

## 3. Results

### 3.1. AD Reduced Weight Loss and Organ Toxicity in STZ-Induced Diabetes

**Figure 3:**
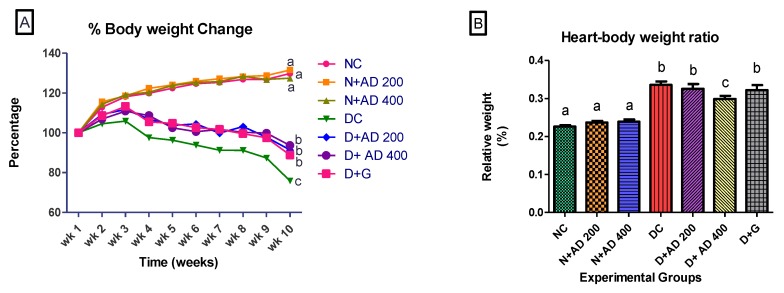
Effect of *Anchomanes difformis* (AD) administration on (**A**) bodyweight change and (**B**) heart–body weight ratio. Points and bars are indicative of mean values ± SEM. Bars with different letters are significantly (*p* < 0.05) different from each other.

Induction of diabetes led to significant reduction in the mean body weight of the diabetic rats (Figure 3A). There was 24.1% weight loss in the untreated diabetic rats, while diabetic rats treated with glibenclamide experienced 11.2% weight loss. Body weight results of the rats treated with 200 mg and 400 mg of AD revealed 8.6% and 6.4% loss in body weight, respectively. Interestingly, both concentrations of AD—400 mg and 200 mg—significantly prevented weight loss by 17.7% and 15.5%, respectively, while glibenclamide reduced weight loss by 12.9%. Both concentrations of AD were able to significantly impede weight loss in STZ-induced diabetes better than the reference drug, glibenclamide (Figure 3A). In addition to the increased weight loss, the heart to body weight ratio of untreated diabetic rats significantly increased by 40% when compared to the normal rats (Figure 3B). Administration of 400 and 200 mg/kg BW AD significantly ameliorated possible hypertrophy by reducing the heart–body weight ratio of diabetic rats by 15% and 11.95%, respectively. Treatment with 5 mg/kg BW of glibenclamide did not reduce relative heart weight in diabetic hearts when compared to the untreated diabetic rats (Figure 3B). This demonstrated the ability of AD to protect against STZ induced organ toxicity.

### 3.2. Effect of AD on Antioxidant Enzymes and Protein Synthesis in STZ Induced Diabetes

**Figure 4:**
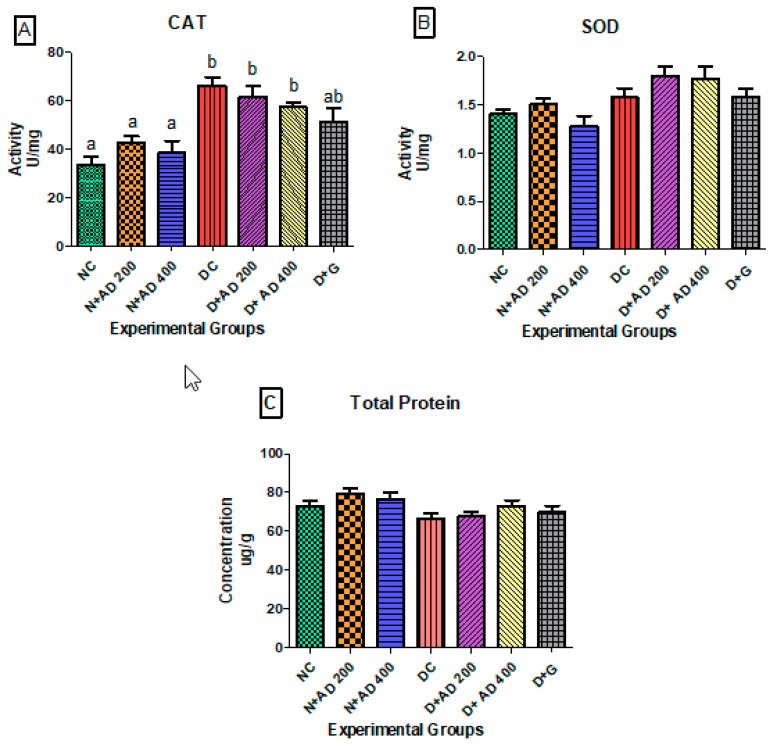
Effect of treatment with AD on the activity of (**A**) catalase (CAT), (**B**) superoxide dismutase (SOD), and (**C**) total protein in the hearts of normal and diabetic rats. Bars are indicative of mean values ± SEM of group values. Bars with different letters are significantly (*p* < 0.05) different from each other; bar with “ab” is not significantly different from bars with “a” or “b”.

Figure 4A,B illustrate the effect of diabetes and administration of AD on catalase and SOD activities in the heart of normal and diabetic rats. Administration of AD led to an observable increase in the activities of catalase and SOD in the normal rats. Also, induction of diabetes by administration of STZ significantly increased the activity of catalase in the hearts of untreated diabetic rats (Figure 4A). However, treatment with AD attempted to revert the abnormally increased levels of catalase, although not significantly. Glibenclamide reduced the catalase levels to near normal. There was no significant difference in the SOD levels in normal and diabetic groups (Figure 4B). A slight decrease in protein levels was observed in the diabetic rats, and the ingestion of AD led to increased protein levels in the treated diabetic rats as presented in Figure 4C; 400 mg/kg BW of AD was able to enhance protein levels in diabetic rats (*p >* 0.05).

### 3.3. Effect of Treatment with AD on Antioxidant Indices in the Hearts of Normal and Diabetic Rats

**Figure 5:**
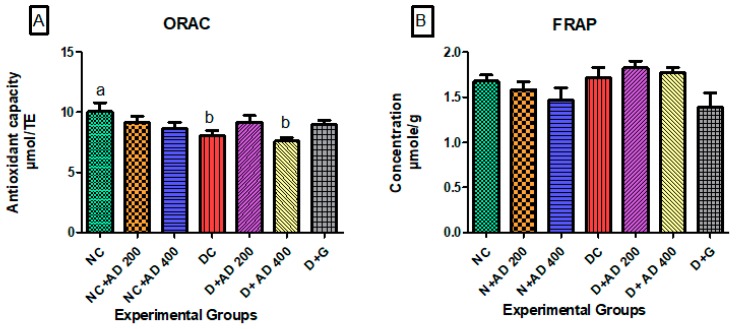
Effect of intervention with AD on the antioxidant capacities; (**A**) ORAC and (**B**) FRAP in the heart of normal and diabetic rats. Bars are indicative of mean values ± SEM of group values. Bars with different letters are significantly (*p* < 0.05) different from each other.

The oxygen radical absorbing ability was significantly reduced in the hearts of untreated diabetic rats when compared to normal rats (Figure 5A). ORAC levels in the diabetic rats administered 200 mg/kg BW and 5 mg/kg BW of AD and glibenclamide, respectively, were comparable to normal. Ferric reducing antioxidant power in the hearts of normal and diabetic rats was not significantly different (Figure 5B).

### 3.4. AD Modulated Hyperglycemia-Induced Immune Response in the Heart in T2D Model

**Figure 6:**
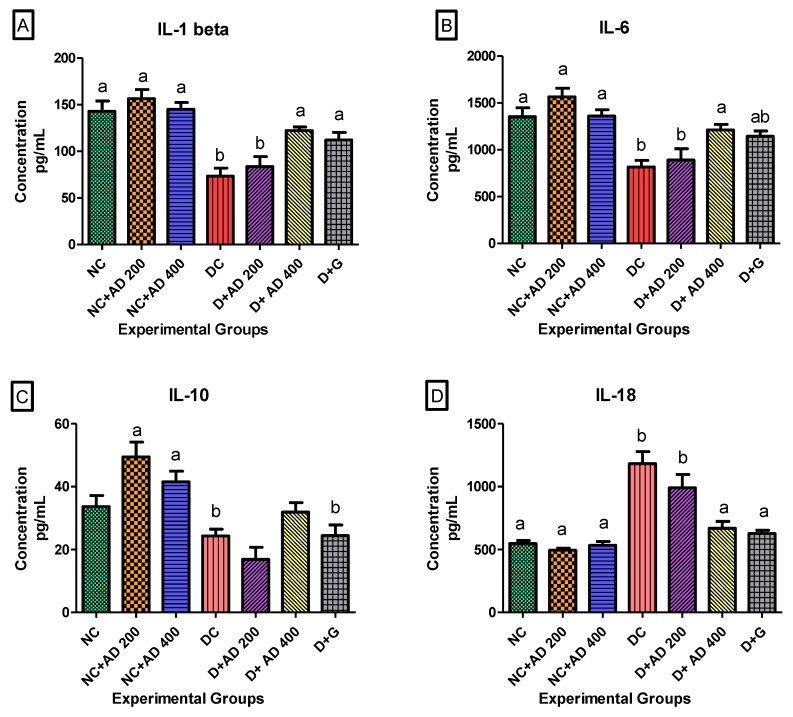
Effect of AD administration on interleukins (IL) (**A**) IL-1β, (**B**) IL-6, (**C**) IL-10, and (**D**) IL-18 in the hearts of normal and diabetic rats. Bars are indicative of mean values ± SEM of group values. Bars with different letters are significantly (*p* < 0.05) different from each other; bar with “ab” is not significantly different from bars with “a” or “b”.

STZ injection significantly increased IL-18 in the hearts of untreated diabetic rats. However, treatment with AD (400 mg/kg BW) and glibenclamide (5 mg/kg BW) restored it to normal levels (Figure 6D). Administration of 200 mg/kg BW of AD caused a 16.2% reduction in IL-18 levels in diabetic hearts but not significantly at *p* < 0.05. The administration of AD increased the concentration of IL-10 in the heart of normal rats, while it significantly decreased in the heart of untreated diabetic rats when compared to normal rats placed on AD (Figure 6C). There was no significant difference in the levels of IL-10 in normal controls and diabetic rats treated with 400 mg/kg BW of AD. IL-6 and IL-1β levels were significantly reduced in the hearts of diabetic rats (untreated and 200 mg/kg BW) when compared with normal rats (Figure 6A,B). This was restored back to normal levels in diabetic rats treated with 400 mg/kg BW of AD and glibenclamide. An increase was observed in the MCP-1 levels in the hearts of untreated rats when compared with normal and treated rats, but this was not significant at 5% level of significance (Table 2). Injection of STZ did not have any significant difference in the TNFα concentrations in the diabetic rats compared to normal rats (Table 2).

### 3.5. The Effect of AD on Lipid Peroxidation and Heart Function Markers

Presence of abnormal concentrations of heart fatty acid binding protein (H-FABP) in the serum is indicative of cardiomyopathy. H-FABP was measured in the serum of normal and diabetic rats. Hyperglycemia caused a significant increase in the levels of H-FABP in the serum of diabetic rats when compared with normal rats (Table 3). Treatment with AD did not have any effect on the increased H-FABP in the serum of treated, diabetic rats. There was no significant difference in the TBARS concentration in normal and diabetic hearts (Table 3).

### 3.6. The Regulation of Transcription Factors by AD in T2D Model

**Figure 7:**
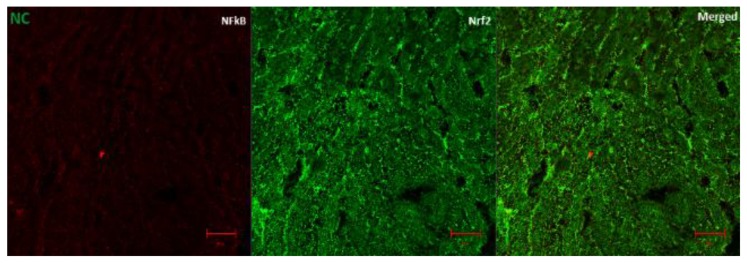

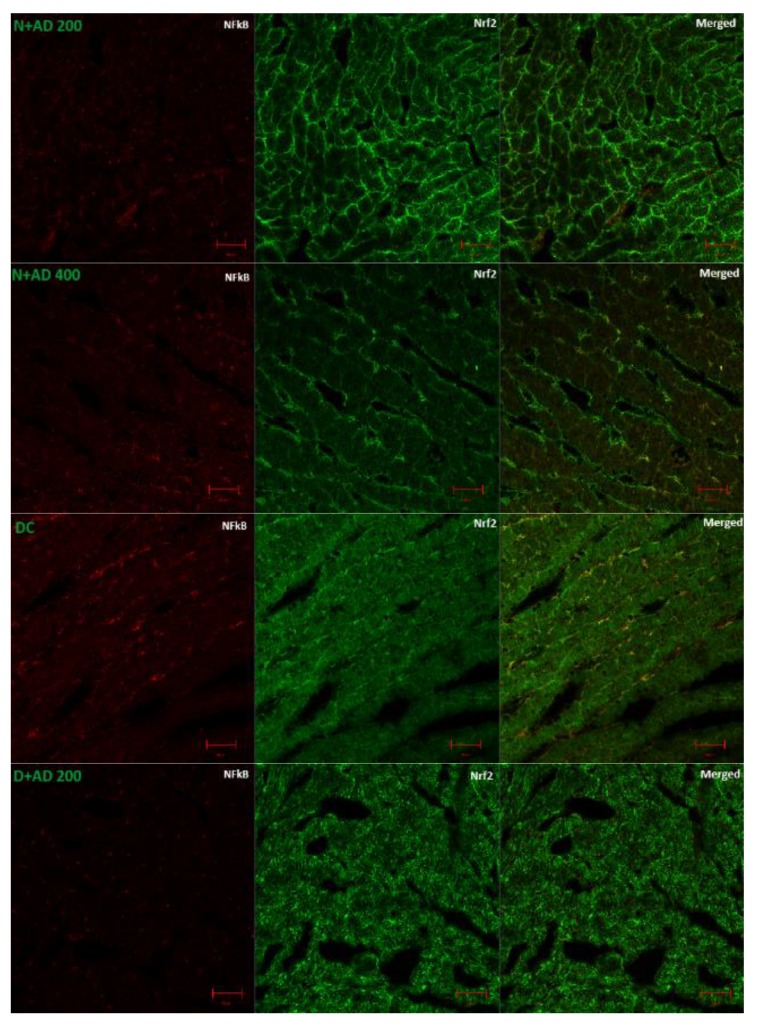

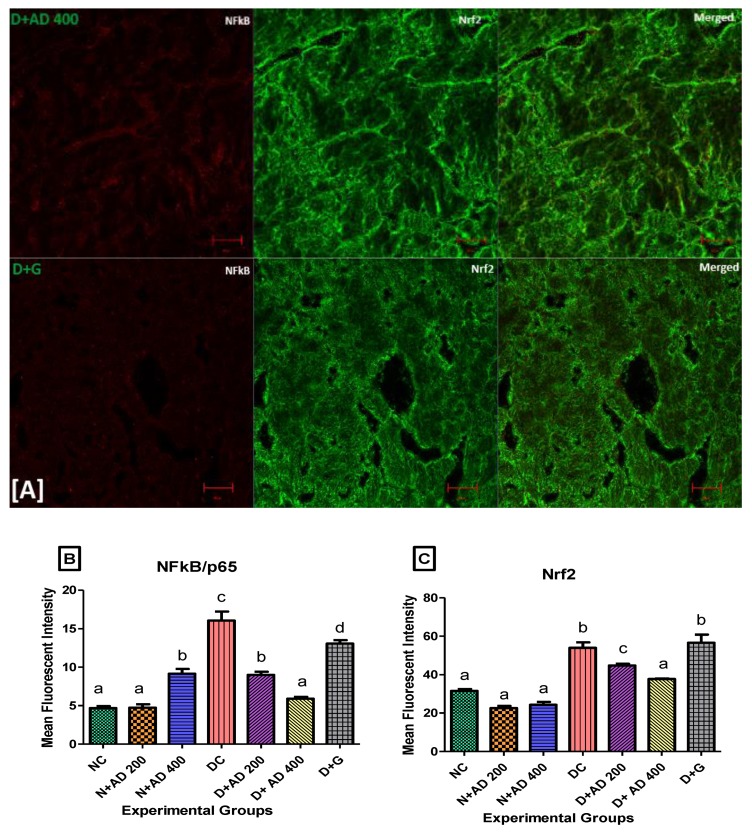
(**A**) Representatives of confocal microscopy image showing the effect of AD on the expression of NFkB/p65 (red) and Nrf2 (green) in the heart tissues. Quantitative analysis of (**B**) NFkB/p65 and (**C**) Nrf2 expression in the heart tissues. Bars are indicative of mean values ± SEM of group values. Bars with different letters are significantly (*p* < 0.05) different from each other.

Figure 7A (fluorescence micrographs) demonstrates the effect of AD intervention on NFkB/p65 and Nrf2 in the hearts of normal and diabetic rats. The expression of Nrf2 and NFkB/p65 was significantly enhanced in the hearts of diabetic rats (Figure 7B,C). This was significantly reduced in the rats treated with 200 and 400 mg/kg BW of AD in a dose dependent manner and comparable to normal rats. Treatment with 5 mg/kg BW of glibenclamide had no effect on the Nrf2 expression in the diabetic hearts, but it significantly reduced the NFkB levels in treated diabetic hearts when compared to diabetic control hearts. However, the reduction of NFkB expressions by glibenclamide was not comparable to normal levels (Figure 7). This increase in Nrf2 and NFkB/p65 expression is suggestive of increased oxidative stress and inflammation in the diabetic rats.

### 3.7. Anti-Apoptotic Effect of AD on T2D Model

**Figure 8:**
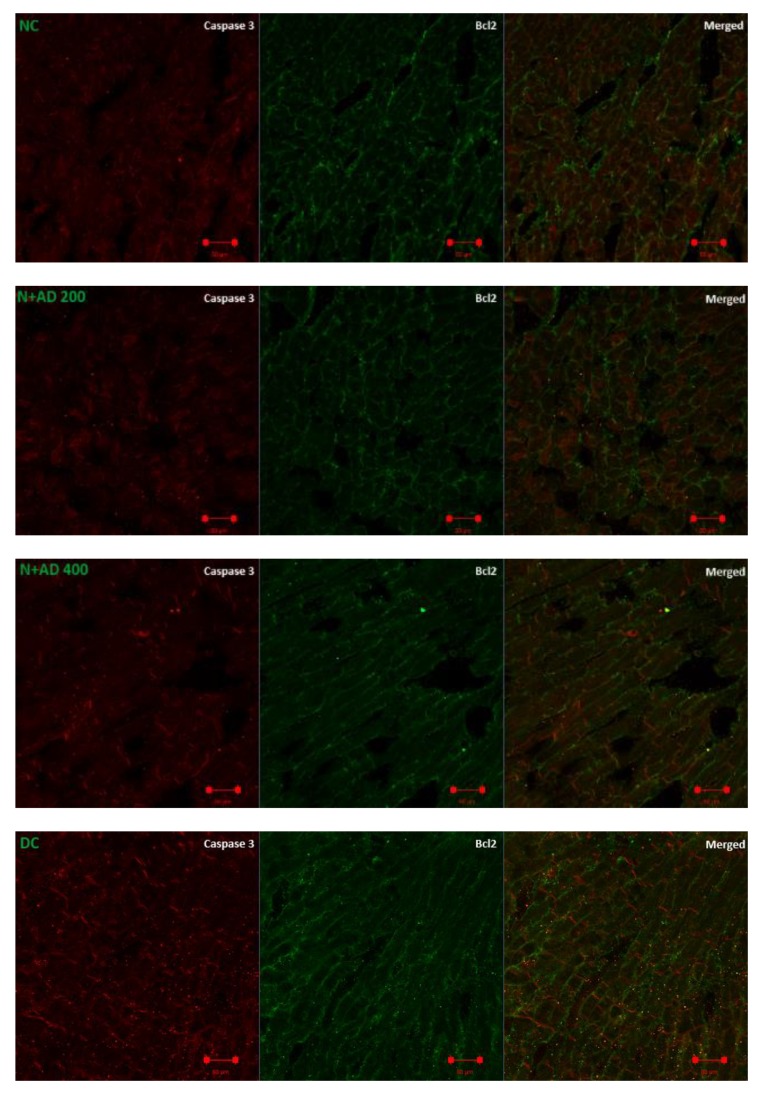

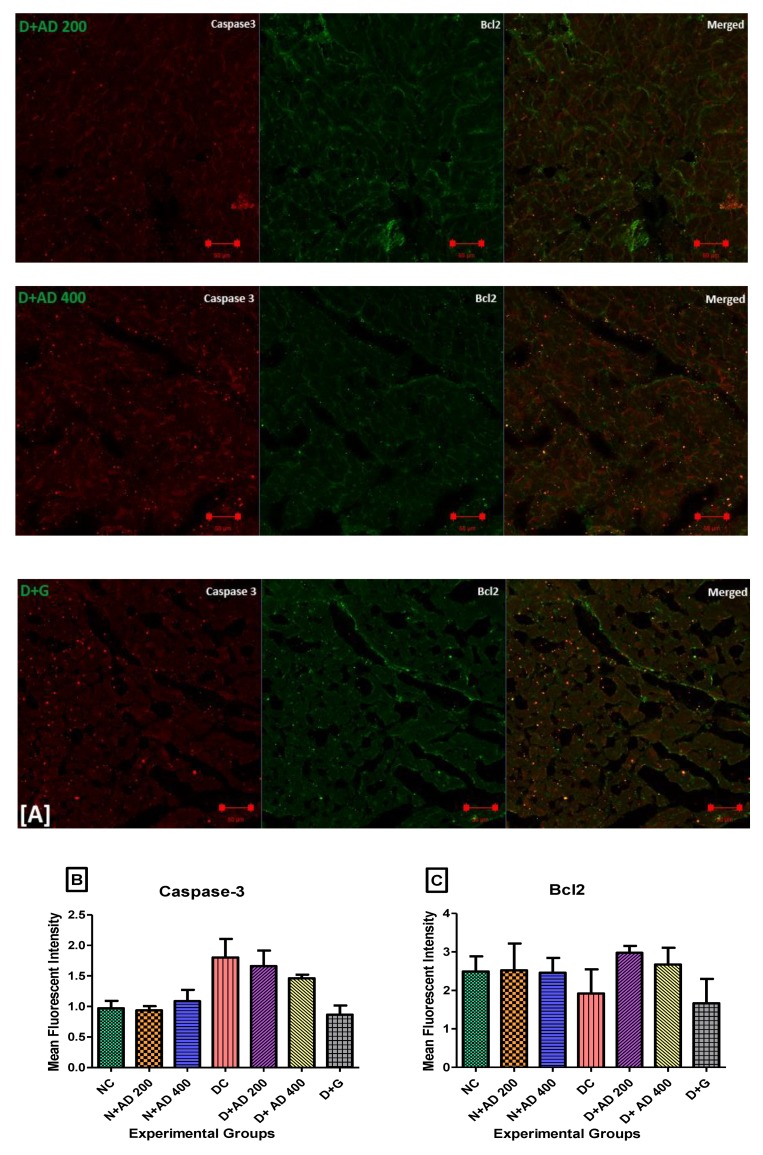
(**A**) Fluorescence micrographs showing the effect of AD intervention on apoptotic markers in the hearts of normal and diabetic rats. (**B**) Quantification of the level of expression of caspase 3 and (**C**) Bcl2 in the heart tissues.

Caspase 3 is a pro-apoptotic marker, while Bcl2 promotes cell survival and inhibits the action of pro-apoptotic markers. The effect of AD administration on caspase 3 and Bcl2 in normal and diabetic rats is displayed in Figure 8. An increase (85.6%) was observed in the expression of caspase 3 in the diabetic hearts, which was not significant at *p* < 0.05 (Figure 8B). Conversely, there was an observable decrease (22.8%) in the levels of Bcl2 in the diabetic hearts when compared with normal rats (Figure 8C). Intervention with 200 and 400 mg/kg BW of AD was able to reduce the expression of caspase 3 by 7.9% and 9.4%; *Bcl2* expression was also upregulated in the diabetic treated rats.

## 4. Discussion

Organ and body weights are essential indices for evaluating the toxicity of a plant or other substances [42]. The administration of STZ and fructose caused a significant body weight loss (24.1%), while intervention with AD significantly prevented weight loss. Rats treated with AD showed between 6.4–8.6% weight loss. Although 5–10% body weight loss has been recommended as an effective strategy for glycemic control in ameliorating diabetes [43], 24.1% weight loss in diabetic control is extreme and implicative of decreased cellular metabolism and growth [44,45]. Also, the loss of insulin sensitivity prevents the utilization of glucose for energy in the cells, thus the body burns fat and muscle as an alternative energy source [46]. Other studies have reported severe weight loss in diabetes mellitus [47,48]. An increase in relative organ weight is indicative of hypertrophy [49]. Hypertrophy combined with other factors—fibrosis, oxidative stress, and apoptosis—in the heart is characteristic of myocardial pathology in diabetic conditions [50]. Treatment with AD was able to significantly ameliorate hypertrophy in diabetic hearts when compared with the diabetic control, which alludes to the cardio-protective effect of AD against the organ impairment and related conditions caused by hyperglycemia. However, the administration of glibenclamide failed to reduce the hypertrophy in the diabetic hearts.

Activation of Nrf2 is one of the pathways initiated during oxidative stress and inflammation to mitigate the overproduction of free radicals. Increased oxidative stress due to hyperglycemia in the diabetic rats resulted in the increased activation and expression of Nrf2 in the diabetic hearts. Interestingly, certain studies reported decreased Nrf2 levels in diabetic cardiomyocytes and other associated vascular conditions [51,52]. However, the mechanism of operation by which Nrf2 is activated shows that it is constitutively expressed in higher amounts in response to oxidative stress, which corroborates our findings [19,53,54]. The expression of Nrf2, which was enhanced in the diabetic hearts, was significantly reduced to near normal levels with AD therapy when compared with the untreated diabetic rats. The ability of AD to normalize Nrf2 levels can be attributed to its inherent antioxidant compounds, which scavenge the free radicals, combatting oxidative stress; hence, the system has lower need to induce the expression of Nrf2.

Reports show that Nrf2 expression enhances the transcription of ARE genes, thereby elevating CAT, SOD, and other antioxidant capacities in the body [55]. The observed increase in the activities of CAT and SOD in this study can be related to the increased expression of Nrf2.

The insignificant decrease in lipid peroxidation in the untreated diabetic rats compared with the treated diabetic and normal rats is indicative of the increased Nrf2 Expression. Studies have shown that Nrf2 inhibits lipid peroxidation and upregulates the transcription of anti-ferroptotic genes, which combats lipid peroxides and prevents ferroptotic cell death [56]. The reduced synthesis of protein in diabetic control is typical of a diabetic condition and can be a result of an increased supply of amino acids for gluconeogenesis [33,57]. Introduction of STZ altered the protein metabolism and led to decreased protein in the diabetic rats. There was no significant difference in the ORAC and the FRAP levels in the hearts of diabetic control, treated, and normal rats. This implies that the diabetic condition and the treatment do not have significant effect on the heart ORAC and FRAP levels.

NFkB, which is activated by increased oxidative stress and hyperglycemia, promotes the transcription and the expression of pro-inflammatory genes and protein in the heart. These cytokines have been linked to pathological conditions [7,14]. NFkB/p65 protein was significantly upregulated in the hearts of diabetic controls; usage of 200 and 400 mg/kg BW of AD significantly diminished NFkB/p65 expression to normal levels in the diabetic hearts. The repressing effect of glibenclamide on NFkB/p65 expression in the diabetic heart is not comparable to AD. This supports ethnopharmacological claims and previous scientific findings of AD as an anti-inflammatory agent.

A relationship has been shown to exist between catalase activities and pro-inflammatory markers such as IL-1β and IL-6. The activity of catalase is inversely proportional to the concentrations of IL-1β and IL-6 [58,59]. Therefore, the significant rise in catalase activities in the untreated diabetic heart led to significantly diminished levels of IL-1β and IL-6. IL-6 can also mediate as an anti-inflammatory agent in local and systemic inflammation [60,61]. Hence, the increase in IL-6 levels in normal and diabetic rats placed on AD can suggestively be attributed to the anti-inflammatory modulation by AD. However, IL-10 concentration significantly declined in the diabetic hearts. Management with AD augments IL-10 in the diabetic heart. In addition, treatment with AD significantly abated the production of IL-18 in diabetic hearts in reference to the diabetic control. Increase of pro-inflammatory markers in the heart has been connected to cardio dysfunction such as impaired left ventricular function [46]. The ability of AD to downregulate IL-18 and upregulate IL-10 signifies its importance in mediating cardiac pathologies associated with diabetic complications.

**Figure 9:**
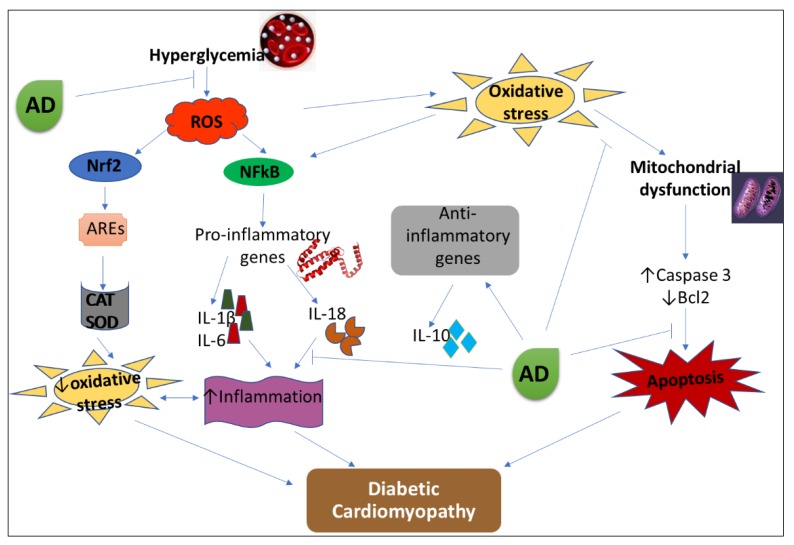
Proposed mechanism of action of AD in the management of DCM. AD-*Anchomanes difformis*, CAT-catalase, SOD-superoxide dismutase, ROS-reactive oxygen species, AREs- antioxidant response elements, Nrf2- nuclear factor E2-related factor 2, NFkB- nuclear factor kappa-light-chain enhancers of activated B cells.

Mitochondrial dysfunction is one of the underlying mechanisms in the progression of DCM, and it is triggered chiefly by oxidative stress leading to distortion in proteins, lipids, and DNA in the mitochondria [62]. Mitochondrial dysfunction further upregulates pro-apoptotic proteins (cytochrome c, caspase 3, -9, -8) and causes increased apoptosis [63]. Increased ROS-mitochondrial mediated apoptosis in the cardiomyocytes is an important factor in the development of various pathologies in diabetic hearts [64]. Bcl2 impedes mitochondrial-mediated apoptosis by blocking activation and release of cytochrome C, which in turn initiates caspase cascades of activation that leads to cell death. Diabetic induction enhanced caspase 3 expression and led to a decline in Bcl2 expression. Supplementation with AD caused an increase in Bcl2 expression and lowered caspase 3 expression in diabetic hearts. This suggests that the ability of AD to moderate apoptosis may be one of the contributing factors in ameliorating DCM. A proposed mechanism of action for AD in ameliorating DCM by modulating apoptosis, inflammation, and oxidative stress is summarized in Figure 9. AD reduced hyperglycemia, inhibited the development of oxidative stress, and was able to retard inflammation and apoptosis in diabetes by blocking the production of IL-18 and caspase 3 via the deregulation of NFkB expressions in the treated diabetic rats.

## 5. Conclusions

The ability of AD to protect against toxicity, reduce inflammation, and mediate apoptosis in the heart presents its potentials to mitigate diabetic cardiomyopathy and hence the need to consider the use of AD as an alternative therapy in prevention and management of diabetic cardiomyopathy.

The ameliorating effect of AD in other diabetic complications such as nephropathy and sexual dysfunction can be explored. In addition, this study can be taken further into clinical trials to assess and validate the therapeutic effect of AD in type 2 diabetes in humans.

## Figures and Tables

**Table 1:** The descriptions of the antibodies used for detection of protein expression levels stating the host, the supplier, and the optimization factor.

No	Marker	Antibody	Host	Source	Dilution
1.	Nrf2	Anti-Nrf2	Mouse	ABCAM, UK	1:200
2.	NFkB/p65	Anti-NFkB/p65	Rabbit	ABCAM, UK	1:250
3.	Bcl2	Anti-Bcl2	Mouse	BioLegend, SA	1:125
4.	Caspase-3	Anti-Caspase-3	Rabbit	ABCAM, UK	1:150
5.	Alexa Fluor 488	Anti-mouse IgG H&L	Goat	ABCAM, UK	1:400
6.	Alexa Fluor 594	Anti-rabbit IgG H&L	Goat	ABCAM, UK	1:400

**Table 2:** The response of tumor necrosis factor alpha (TNFα) and monocyte chemoattractant protein 1 (MCP-1) to treatment with AD in normal and diabetic hearts.

Experimental Groups	TNFα (pg/mL)	MCP-1 (pg/mL)
NC	56.87 ± 3.68	288.8 ± 19.34
N + AD 200	61.39 ± 2.42	272.1 ± 23.34
N + AD 400	60.05 ± 3.38	287.4 ± 15.24
DC	50.40 ± 3.06	306.3 ± 15.03
D + AD 200	48.50 ± 3.68	280.7 ± 12.25
D + AD 400	49.72 ± 2.28	301.1 ± 23.00
D + G	51.52 ± 3.06	313.1 ± 27.05

Normal control (NC) received the vehicle only. Animals in groups 2 and 3 were normal rats who received 200 and 400 mg/kg BW of AD aqueous extract only (N + AD 200 and N + AD 400), and these served as the treated control. Groups 4–7 consisted of animals that were placed on 10% fructose for 2 weeks followed by streptozotocin (STZ). Group 4 rats received vehicle only (DC), rats in groups 5 and 6 were given 200 and 400 mg/kg BW of AD aqueous extract (D + AD 200 and D + AD 400), respectively, while group 7 rats received 5 mg/kg BW of glibenclamide, an antidiabetic drug (D + G).

**Table 3:** The effect of AD on (**A**) heart-type fatty acid-binding protein (H-FABP) and (**B**) thiobarbituric acid reactive substances (TBARS) levels in the hearts of normal and diabetic hearts.

Experimental Groups	H-FABP (ng/mL)	TBARS (pmole/g)
NC	0.216 ± 0.011 ^a^	2.226 ± 0.15
N + AD 200	0.2784 ± 0.004	2.080 ± 0.09
N + AD 400	0.2896 ± 0.003	2.032 ± 0.16
DC	0.2955 ± 0.002 ^b^	1.809 ± 0.10
D + AD 200	0.2926 ± 0.006 ^b^	2.068 ± 0.11
D + AD 400	0.2918 ± 0.005 ^b^	2.371 ± 0.10
D + G	0.3121 ± 0.003 ^b^	1.867 ± 0.12

Values with different letters are significantly (*p* < 0.05) different from each other.

## Abbreviations

AD	*Anchomanes difformis*
ARE	Antioxidant response elements
Bcl2	B-cell lymphoma 2
CAT	Catalase
FRAP	Ferric reducing antioxidant power
H-FABP	Heart fatty acid binding protein
IL	Interleukin
KEAP-1	Kelch-like ECH-associated protein 1
MDA	Malondialdehyde
MCP-1	Monocyte chemoattractant protein 1
Nrf2	Nuclear factor-erythroid 2-related factor 2
NFkB	Nuclear factor kappa-light-chain-enhancer of activated B cells
ORAC	Oxygen radical absorbance capacity
PBS	Phosphate buffer saline
ROS	Reactive oxygen species
SOD	Superoxide dismutase
STZ	Streptozotocin
TBARS	Thiobarbituric acid reactive substances
TNF-α	Tumor necrosis factor alpha

## References

[1] Miranda-Díaz A.G., Pazarín-Villaseñor L., Yanowsky-Escatell F.G., Andrade-Sierra J. (2016). Oxidative Stress in Diabetic Nephropathy with Early Chronic Kidney Disease. J. Diabetes Res..

[2] Althunibat O.Y., al Hroob A.M., Abukhalil M.H., Germoush M.O., Bin-Jumah M., Mahmoud A.M. (2019). Fisetin ameliorates oxidative stress, inflammation and apoptosis in diabetic cardiomyopathy. Life Sci..

[3] Li L., Luo W., Qian Y., Zhu W., Qian J., Li J., Jin Y., Xu X., Liang G. (2019). Luteolin protects against diabetic cardiomyopathy by inhibiting NF-κB-mediated inflammation and activating the Nrf2-mediated antioxidant responses. Phytomedicine.

[4] Al Hroob A.M., Abukhalil M.H., Hussein O.E., Mahmoud A.M. (2019). Pathophysiological mechanisms of diabetic cardiomyopathy and the therapeutic potential of epigallocatechin-3-gallate. Biomed. Pharmacother..

[5] Paolillo S., Marsico F., Prastaro M., Renga F., Esposito L., de Martino F., di Napoli P., Esposito I., Ambrosio A., Ianniruberto M. (2019). Diabetic Cardiomyopathy. Heart Fail. Clin..

[6] Bajpai A., Tilley D.G. (2018). The Role of Leukocytes in Diabetic Cardiomyopathy. Front. Physiol..

[7] Nunes S., Rolo A.P., Palmeira C.M., Reis F. (2017). Diabetic Cardiomyopathy: Focus on Oxidative Stress, Mitochondrial Dysfunction and Inflammation. Cardiomyopathies—Types Treat.

[8] Zhang H., Sun S.-C. (2015). NF-κB in inflammation and renal diseases. Cell Biosci..

[9] Liu T., Zhang L., Joo D., Sun S.-C. (2017). NF-κB signaling in inflammation, Signal Transduct. Target. Ther..

[10] Mano Y., Anzai T., Kaneko H., Nagatomo Y., Nagai T., Anzai A., Maekawa Y., Takahashi T., Meguro T., Yoshikawa T. (2011). Overexpression of Human C-Reactive Protein Exacerbates Left Ventricular Remodeling in Diabetic Cardiomyopathy. Circ. J..

[11] Yu X.-Y., Chen H.-M., Liang J.-L., Lin Q.-X., Tan H.-H., Fu Y.-H., Liu X.-Y., Shan Z.-X., Li X.-H., Yang H.-Z. (2011). Hyperglycemic Myocardial Damage Is Mediated by Proinflammatory Cytokine: Macrophage Migration Inhibitory Factor. PLoS ONE.

[12] Fuentes-Antrás J., Ioan A.M., Tuñón J., Egido J., Lorenzo Ó. (2014). Activation of Toll-Like Receptors and Inflammasome Complexes in the Diabetic Cardiomyopathy-Associated Inflammation. Int. J. Endocrinol..

[13] Goldin A., Beckman J.A., Schmidt A.M., Creager M.A. (2006). Advanced Glycation End Products. Circulation.

[14] Nishida K., Otsu K. (2017). Inflammation and metabolic cardiomyopathy. Cardiovasc. Res..

[15] Kiraz Y., Adan A., Yandim M.K., Baran Y. (2016). Major apoptotic mechanisms and genes involved in apoptosis. Tumor Biol..

[16] Redza-dutordoir M., Averill-bates D.A. (2016). Biochimica et Biophysica Acta Activation of apoptosis signalling pathways by reactive oxygen species. BBA Mol. Cell Res..

[17] Nunes S., Soares E., Pereira F., Reis F. (2012). The role of inflammation in diabetic cardiomyopathy. Int. J. Infereron Cytokine Mediat. Res..

[18] Ma Q. (2013). Role of Nrf2 in Oxidative Stress and Toxicity. Annu. Rev. Pharmacol. Toxicol..

[19] Hong F., Sekhar K.R., Freeman M.L., Liebler D.C. (2005). Specific Patterns of Electrophile Adduction Trigger Keap1 Ubiquitination and Nrf2 Activation. J. Biol. Chem..

[20] Kobayashi M., Yamamoto M. (2006). Nrf2–Keap1 regulation of cellular defense mechanisms against electrophiles and reactive oxygen species. Adv. Enzyme Regul..

[21] Baird L., Dinkova-Kostova A.T. (2011). The cytoprotective role of the Keap1–Nrf2 pathway. Arch. Toxicol..

[22] Merchant A.A., Singh A., Matsui W., Biswal S. (2011). The redox-sensitive transcription factor Nrf2 regulates murine hematopoietic stem cell survival independently of ROS levels. Blood.

[23] Hennig P., Garstkiewicz M., Grossi S., di Filippo M., French L., Beer H.-D. (2018). The Crosstalk between Nrf2 and Inflammasomes. Int. J. Mol. Sci..

[24] Eleazu C., Eleazu K., Chukwuma S., Essien U. (2013). Review of the mechanism of cell death resulting from streptozotocin challenge in experimental animals, its practical use and potential risk to humans. J. Diabetes Metab. Disord..

[25] Furman B.L. (2015). Streptozotocin-Induced Diabetic Models in Mice and Rats. Curr. Protoc. Pharmacol..

[26] Ma M., Mu T. (2016). Anti-diabetic effects of soluble and insoluble dietary fibre from deoiled cumin in low-dose streptozotocin and high glucose-fat diet-induced type 2 diabetic rats. J. Funct. Foods.

[27] Barrière D.A., Noll C., Roussy G., Lizotte F., Kessai A., Kirby K., Belleville K., Beaudet N., Longpré J.-M., Carpentier A.C. (2018). Combination of high-fat/high-fructose diet and low-dose streptozotocin to model long-term type-2 diabetes complications. Sci. Rep..

[28] Wilson R.D., Islam M.S. (2012). Fructose-fed streptozotocin-injected rat: An alternative model for type 2 diabetes. Pharmacol. Rep..

[29] Udje T.D., Brooks N.L., Oguntibeju Oluwafemi O., Goyal M.R., Ayeleso A.O. (2018). Medicinal Activities of Anchomanes difformis and its Potential in the Treatment of Diabetes Mellitus and Other Disease Conditions: A Review. Bioactive Compounds of Medicinal Plants.

[30] Agyare C., Boakye Y.D., Apenteng J.A., Dapaah S.O., Appiah T., Adow A. (2015). Antimicrobial and Anti-Inflammatory Properties of *Anchomanes difformis* (Bl.) Engl. and *Colocasia esculenta* (L.) Schott. Biochem. Pharmacol. Open Access..

[31] Adebayo A.H., John-Africa L.B., Agbafor A.G., Omotosho O.E., Mosaku T.O. (2014). Anti-nociceptive and anti-inflammatory activities of extract of Anchomanes difformis in rats. Pak. J. Pharm. Sci..

[32] Aderonke S.O., Ezinwanne A.J. (2015). Evaluation of the Anti Diabetic Activity of Ethanol Extract of Anchomanes Difformis (Araceae) Leaves in Albino Rats. Int. Res. J. Pharm..

[33] Adeyemi O., Makinwa T.T., Uadia R.N. (2015). Ethanol Extracts of Roots of Anchomanes difformis ENGL Roots as an Antihyperglycemic Agent in Diabetic Rats. Chem. J..

[34] Alabi T.D., Brooks N.L., Oguntibeju O.O. (2019). Antioxidant Capacity, Phytochemical Analysis and Identification of Active Compounds in Anchomanes difformis. Nat. Prod. J..

[35] Najafian M., Jahromi M.Z., Nowroznejhad M.J., Khajeaian P., Kargar M.M., Sadeghi M., Arasteh A. (2012). Phloridzin reduces blood glucose levels and improves lipids metabolism in streptozotocin-induced diabetic rats. Mol. Biol. Rep..

[36] Josef R., Giribabu N., Karim K., Salleh N. (2017). Quercetin ameliorates oxidative stress, inflammation and apoptosis in the heart of streptozotocin-nicotinamide-induced adult male diabetic rats. Biomed. Pharmacother..

[37] Ellerby L.M., Bredesen D.E. (2000). Measurement of cellular oxidation, reactive oxygen species, and antioxidant enzymes during apoptosis. Methods Enzymol..

[38] Matsunami T., Sato Y., Sato T., Yukawa M. (2010). Antioxidant status and lipid peroxidation in diabetic rats under hyperbaric oxygen exposure. Physiol. Res..

[39] Wasowicz W., Neve J., Peretz A. (1993). Optimized steps in fluorometric determination of thiobarbituric acid-reactive substances in serum: Importance of extraction pH and influence of sample preservation and storage. Clin. Chem..

[40] Prior R.L., Hoang H.A., Gu L., Wu X., Bacchiocca M., Howard L., Hampsch-Woodill M., Huang D., Ou B., Jacob R. (2003). Assays for hydrophilic and lipophilic antioxidant capacity (oxygen radical absorbance capacity (ORACFL)) of plasma and other biological and food samples. J. Agric. Food Chem..

[41] Benzie I.F., Strain J.J. (1999). Ferric reducing/antioxidant power assay: Direct measure of total antioxidant activity of biological fluids and modified version for simultaneous measurement of total antioxidant power and ascorbic acid concentration. Methods in Enzymology.

[42] Akhigbe R.E. (2014). Discordant Results in Plant Toxicity Studies in Africa: Attempt of Standardization. Toxicological Survey of African Medicinal Plants.

[43] Delahanty L.M. (2017). Weight loss in the prevention and treatment of diabetes. Prev. Med..

[44] Hispard F., de Vaufleury A., Martin H., Devaux S., Cosson R.P., Scheifler R., Richert L., Berthelot A., Badot P.-M. (2008). Effects of subchronic digestive exposure to organic or inorganic cadmium on biomarkers in rat tissues. Ecotoxicol. Environ. Saf..

[45] Ige S.F., Akhigbe R.E., Edeogho O., Ajao F.O., Owolabi O.Q., Oyekunle O.S., Ajayi A.F. (2011). Hepatoprotective activities of Allium cepa in cadmium-treated rats. Int. J. Pharm. Pharm. Sci..

[46] Khanra R., Dewanjee S., Dua T.K., Sahu R., Gangopadhyay M., DeFeo V., Zia-Ul-Haq M. (2015). *Abroma augusta* L. (Malvaceae) leaf extract attenuates diabetes induced nephropathy and cardiomyopathy via inhibition of oxidative stress and inflammatory response. J. Transl. Med..

[47] Mátyás C., Németh B.T., Oláh A., Török M., Ruppert M., Kellermayer D., Barta B.A., Szabó G., Kökény G., Horváth E.M. (2017). Prevention of the development of heart failure with preserved ejection fraction by the phosphodiesterase-5A inhibitor vardenafil in rats with type 2 diabetes. Eur. J. Heart Fail..

[48] Addepalli V., Suryavanshi S.V. (2018). Catechin attenuates diabetic autonomic neuropathy in streptozotocin induced diabetic rats. Biomed. Pharmacother..

[49] Al-Malki A.L., el Rabey H.A. (2015). The Antidiabetic Effect of Low Doses of Moringa oleifera Lam. Seeds on Streptozotocin Induced Diabetes and Diabetic Nephropathy in Male Rats. Biomed. Res. Int..

[50] Huynh K., Bernardo B.C., McMullen J.R., Ritchie R.H. (2014). Diabetic cardiomyopathy: Mechanisms and new treatment strategies targeting antioxidant signaling pathways. Pharmacol. Ther..

[51] Tan Y., Ichikawa T., Li J., Si Q., Yang H., Chen X., Goldblatt C.S., Meyer C.J., Li X., Cai L. (2011). Diabetic downregulation of Nrf2 activity via ERK contributes to oxidative stress-induced insulin resistance in cardiac cells in vitro and in vivo. Diabetes.

[52] Lu T., Sun X., Li Y., Chai Q., Wang X.L., Lee H.C. (2017). Role of Nrf2 signaling in the regulation of vascular BK channel β1 subunit expression and BK channel function in high-fat diet–induced diabetic mice. Diabetes.

[53] Li B., Liu S., Miao L., Cai L. (2012). Prevention of Diabetic Complications by Activation of Nrf2: Diabetic Cardiomyopathy and Nephropathy. Exp. Diabetes Res..

[54] Kobayashi A., Kang M.-I., Watai Y., Tong K.I., Shibata T., Uchida K., Yamamoto M. (2006). Oxidative and Electrophilic Stresses Activate Nrf2 through Inhibition of Ubiquitination Activity of Keap1. Mol. Cell. Biol..

[55] Benipal S.S., Liu T., Knowlton A. (2017). Repetitive ROS Inhibits Nrf2 Antioxidant Defense in Ischemic Heart Failure. FASEB J..

[56] Dodson M., Castro-Portuguez R., Zhang D.D. (2019). NRF2 plays a critical role in mitigating lipid peroxidation and ferroptosis. Redox Biol..

[57] Hasan H.R., Abdulsattar A. (2015). Influence of diabetes disease on concentration of total protein, albumin and globulins in saliva and serum: A comparative study. Iraqi Natl. J. Chem..

[58] Mathy-Hartert M., Hogge L., Sanchez C., Deby-Dupont G., Crielaard J.M., Henrotin Y. (2008). Interleukin-1β and interleukin-6 disturb the antioxidant enzyme system in bovine chondrocytes: A possible explanation for oxidative stress generation. Osteoarthr. Cartil..

[59] Gutierrez-Ruiz M.C., Quiroz L.E.G., Hernandez E., Bucio L., Souza V., Llorente L., Kershenobich D. (2001). Cytokine response and oxidative stress produced by ethanol, acetaldehyde and endotoxin treatment in HepG2 cells. Isr. Med. Assoc. J..

[60] Del Giudice M., Gangestad S.W. (2018). Rethinking IL-6 and CRP: Why they are more than inflammatory biomarkers, and why it matters. Brain. Behav. Immun..

[61] Schett G. (2018). Physiological effects of modulating the interleukin-6 axis. Rheumatology.

[62] Bhat A.H., Dar K.B., Anees S., Zargar M.A., Masood A., Sofi M.A., Ganie S.A. (2015). Oxidative stress, mitochondrial dysfunction and neurodegenerative diseases; a mechanistic insight. Biomed. Pharmacother..

[63] Nakamura H., Matoba S., Iwai-Kanai E., Kimata M., Hoshino A., Nakaoka M., Katamura M., Okawa Y., Ariyoshi M., Mita Y. (2012). p53 Promotes Cardiac Dysfunction in Diabetic Mellitus Caused by Excessive Mitochondrial Respiration-Mediated Reactive Oxygen Species Generation and Lipid Accumulation. Circ. Heart Fail..

[64] Boudina S., Abel E.D. (2007). Diabetic Cardiomyopathy Revisited. Circulation.

